# Microbial Removal of Heavy Metals from Contaminated Environments Using Metal-Resistant Indigenous Strains

**DOI:** 10.3390/jox14010004

**Published:** 2023-12-29

**Authors:** Cristina Firincă, Lucian-Gabriel Zamfir, Mariana Constantin, Iuliana Răut, Luiza Capră, Diana Popa, Maria-Lorena Jinga, Anda Maria Baroi, Radu Claudiu Fierăscu, Nicoleta Olguța Corneli, Carmen Postolache, Mihaela Doni, Ana-Maria Gurban, Luiza Jecu, Tatiana Eugenia Șesan

**Affiliations:** 1 Biotechnology and Bioanalysis Departments, National Institute of Research and Development in Chemistry and Petrochemistry—ICECHIM, 202 Spl. Independenței, 060021 Bucharest, Romania; cristina.firinca@icechim.ro (C.F.); mihaela.doni@icechim.ro (M.D.); 2 Department of Botany and Microbiology, Faculty of Biology, University of Bucharest, 91–95 Spl. Independenței, 050095 Bucharest, Romania; 3 Department of Pharmacy, Faculty of Pharmacy, University Titu Maiorescu of Bucharest, 040441 Bucharest, Romania; 4 National Institute of Research and Development for Microbiology and Immunology—Cantacuzino, 103 Spl. Independenței, 050096 Bucharest, Romania; 5 Field Crop Section, Academy of Agricultural and Forestry Sciences, Bd Mărăști 61, 011464 Bucharest, Romania

**Keywords:** soil bioremediation, heavy metals, microorganisms, nanomaterials

## Abstract

Contamination of soil with heavy metals has become a matter of global importance due to its impact on agriculture, environmental integrity, and therefore human health and safety. Several microbial strains isolated from soil contaminated by long-term chemical and petrochemical activities were found to manifest various levels of tolerance to Cr, Pb, and Zn, out of which *Bacillus marisflavi* and *Trichoderma longibrachiatum* exhibited above-moderate tolerance. The concentrations of target heavy metals before and after bioremediation were determined using electrochemical screen-printed electrodes (SPE) modified with different nanomaterials. The morpho-structural SEM/EDX analyses confirmed the presence of metal ions on the surface of the cell, with metal uptake being mediated by biosorption with hydroxyl, carboxyl, and amino groups as per FTIR observations. *T. longibrachiatum* was observed to pose a higher bioremediation potential compared to *B. marisflavi*, removing 87% of Cr and 67% of Zn, respectively. Conversely, *B. marisflavi* removed 86% of Pb from the solution, compared to 48% by *T. longibrachiatum*. Therefore, the fungal strain *T. longibrachiatum* could represent a viable option for Cr and Zn bioremediation strategies, whereas the bacterial strain *B. marisflavi* may be used in Pb bioremediation applications.

## 1. Introduction

The status of environmental degradation has worsened over the last decades as a consequence of urbanization and industrialization, correlated with the generation of solid, liquid, and gaseous wastes. Recalcitrant pollutants, such as volatile organic compounds, heavy metals, pesticides, hydrocarbons, persistent organic pollutants, solvents, dyes, and plastics, can pose adverse effects on health [1,2,3,4]. Anthropogenic activities such as smelting, mining, fossil fuel refining, the application of agrochemicals, and the disposal of municipal wastes are the primary causes of the accumulation of heavy metals in soils and other environmental compartments [5,6,7]. Heavy metal contamination determines significant modifications to soil processes [8], the microbial community [9], plants [10], and animals [11].

Based on their physiological functionality, heavy metals may be essential (Zn^2+^, Mn^2+^, Fe^2+^, Cu^2+^, Co^2+^, Mo^2+^), playing a vital role in trace amounts in numerous metabolic processes, or non-essential (Pb^2+^, Hg^2+^, As^3+^, Cd^2+^, Cr^6+^), having no contribution to physiological and biochemical functions [12,13]. Both categories pose different levels of toxicity, depending on the concentration and duration of exposure [14]. The toxicity takes effect through inflammation and oxidative stress, which ultimately cause DNA damage due to the accumulation of reactive oxygen species (ROS) [15,16]. Lead has been commonly used for a wide range of products and industrial processes due to its malleability, ductility, and ease of being molded into alloys with other metals [17]. Zinc is the fourth most used metal for production purposes worldwide, along with iron, aluminum, and copper, having various applications in the chemical, pharmaceutical, agricultural, and automotive industries [18]. In the case of chromium, areas of application include electroplating, the production of stainless steel and nonferrous alloys, wood preservation, tanning, and the production of pigments and anti-corrosives [19]. The main detoxification processes employed by microorganisms in the presence of these heavy metals include uptake through functional groups present in the structure of the cellular wall and specific uptake systems [20], the synthesis of enzymes to reduce the pollutants to less toxic forms, such as the case for chromate reductase involved in the reduction of Cr(VI) to Cr(III) [21], complexation, volatilization, and intracellular sequestration [22].

Numerous bacterial species such as *Bacillus*, *Pseudomonas*, *Micrococcus*, and *Enterobacter* [23] and fungal species such as *Penicillium*, *Aspergillus* [24], Fusarium, *Trichoderma*, and *Alternaria* [25] have been studied for their bioremediation potential in heavy metal-contaminated environments. Conventional methods for treating environments contaminated with heavy metals and other toxic elements include physical, chemical, and biological approaches [26]. Physical methods seek to immobilize or mechanically extract pollutants to isolate the contaminated site and prevent further dispersion and contamination [27], whereas chemical methods utilize various reagents, even nanomaterials, to reduce the mobility, toxicity, and bioavailability of pollutants [28]. Both physical and chemical methods require high costs and affect the physico-chemical structure and environmental functions of the soil [29]. Furthermore, the wastes resulting from the treatments, along with the dispersion of fine particles during the displacement of soil for ex situ remediation, pose a significant risk of secondary pollution [30]. Biological methods seek to reduce the bioavailability and toxicity of pollutants by using microorganisms, plants, and algae, thus having the potential to be both economically and ecologically favorable, as well as more accepted by the public, aiding in ecological restoration [30]. 

Numerous studies have demonstrated the efficiency of microorganisms in reducing the concentration and toxicity of various heavy metals [31,32,33,34,35]. Long-term exposure generates modifications in species richness and diversity, with metal-resistant strains becoming dominant, albeit fewer in number [36]. Non-native microbial communities are in competition with the native microbiota and face difficulties in surviving in a polluted environment [37], whereas native strains develop tolerance over time through several extracellular and intracellular mechanisms, thus being more efficient in bioremediation [38,39,40]. Pollution-induced community tolerance (PICT) was proposed for the first time in 1988 by Blanck et al. [41], which suggests that microbial communities exposed to xenobiotic compounds for a sufficient period of time could develop selective resistance as well as suffer modifications in species composition. In that sense, indigenous microbial strains have a higher bioremediation potential for pollutants compared to non-indigenous strains. In terms of determining the most efficient microbial strains for bioremediation, studies that compare the potential of bacteria and fungi are scarce. Also, there is a need to optimize the process in order to be applicable in situ. Furthermore, accessible detection methods for in-field monitoring of the efficiency of bioremediation treatments are required for the improvement of ecological restoration programs.

In Romania, the economic sectors with a significant contribution to heavy metal pollution are the mining and metallurgical industry, chemical production, and improper management of domestic and industrial wastes [42]. Therefore, the objectives of the current study were: (i) to isolate metal-tolerant indigenous microorganisms from soil contaminated with heavy metals; (ii) to test their efficiency in reducing the concentration of Cr, Pb, and Zn; and (iii) to compare the bioremediation potential exhibited by bacterial and fungal isolates. This is a complex study that aims to gain an understanding of the bioremediation process and ensure the monitoring and control of these heavy metals in various polluted environments, such as soil and waters, using portable, simple, and affordable (bio)analytical tools. Nanomaterials-based electrochemical sensors were used for fast and simple determination of the concentration of heavy metals in the samples before and after bioremediation, assessing in this way the efficiency of the process.

## 2. Materials and Methods

### 2.1. Reagents

Tris hydrochloride (Tris-HCl), potassium chloride (KCl), hydrochloric acid (HCl), low molecular weight chitosan (CS, MW = 50–190 kDa), potassium dichromate (K_2_Cr_2_O_7_), lead (II) nitrate Pb(NO_3_)_2_, zinc sulfate heptahydrate (ZnSO_4_·7H_2_O), potassium ferricyanide (K_4_Fe(CN)_6_), ferric chloride (FeCl_3_), tetrachloroauric(III) acid trihydrate, and sodium citrate were obtained from Sigma-Aldrich, Merck KGaA, Darmstadt, Germany. The multi-walled carbon nanotubes (MWCNT) were purchased from Carbon GmbH, Bayreuth, Germany. The electrochemical measurements for chromium and zinc were carried out in a solution containing 0.1 M HCl and 0.1 M KCl, and those for lead in a 0.1 M Tris-HCl buffer pH 5, respectively. All solutions used in the experiments were prepared using ultrapure water (18.25 MΩ·cm) obtained with a Milli-Q^®^ Integral 5 system, Merck KGaA, Darmstadt, Germany.

Analytical grade HNO_3_ 65%, HCl 37%, and HF 50% for the acid digestion of soil samples were obtained from Thermo Fisher Scientific, Waltham, MA, USA. Standard solutions for chromium, lead, and zinc were obtained from Merck, KGaA, Darmstadt, Germany. NaCl, as well as dehydrated culture media, namely PDA, TSA, PDB, TSB, Luria–Bertani, and Sabouraud, were obtained from Scharlau, Sentmenat, Spain.

### 2.2. Sample Collection

Soil samples were collected from Bucharest, Romania (44°26′06″ N 26°11′29″ E), in March 2023. The study area was characterized by a history of research and production in chemistry and petrochemistry between 1986 and 1997, as well as intense traffic and construction activities to date. At the time of sampling, vegetable cover was poorly developed, with scarce grassland patches. Four composite samples consisting of five subsamples were collected from random points over a surface of 200 m^2^ from the surface horizon at a depth of 5 cm. Sterile plastic utensils were used, and the samples were further stored in sterile autoclave bags to be transported to the laboratory for further processing. Samples were sieved through a 2 mm sieve and stored at 4 °C for the duration of the studies.

### 2.3. Elemental Analysis of Soil Samples

The heavy metal profile of the soil was determined using an Optima 2100 DV ICP-OES System (Perkin Elmer, Waltham, MA, USA) equipped with a dual-view optical system. Dried and triturated soil in a quantity of 0.15 g was digested with HNO_3_, HCl, and HF (6:2:2) in a microwave digestion system (Multiwave 3000, Anton Parr GmbH, Graz, Austria). The obtained solutions were subsequently filtered and diluted with ultrapure water. Calibration curves for Cr, Pb, and Zn were constructed from Certipur standard solutions for each element. 

### 2.4. Isolation and Identification of Microorganisms from the Contaminated Soil

Bacterial and fungal strains were isolated through serial dilutions. Samples consisting of 5 g of soil were suspended in 45 mL of physiologic sterile water (0.85% NaCl) and homogenized by incubating in a rotary shaker for 30 min at 170 rpm. Aliquots of 100 μL were subsequently spread aseptically onto agar plates supplemented with 10 mg/L of K_2_Cr_2_O_7_, ZnSO_4_, and Pb (NO_3_)_2_, which were further incubated for up to 7 days at 37° ± 1 °C (bacteria) and 28° ± 1 °C (fungi), respectively. Two different culture media were used for the growth and preservation of the isolated microorganisms: potato dextrose agar (PDA), consisting of (g/L): 4, potato peptone; 20, glucose; 15, agar, for fungal strains; and tryptic soy agar (TSA), consisting of (g/L): 20, peptone; 5, NaCl; 2.5, dipotassium phosphate (K_2_HPO_4_); 2.5, dextrose; 15, agar, for bacterial strains.

Morphologically different colonies were further selected and purified by streaking repeatedly on agar media without metal salts. Characterization was carried out by Gram staining for bacteria and Lactophenol Cotton Blue staining for fungi, followed by microscopic observation using an Olympus BX51 optical microscope. 

For the identification of the isolated microorganisms, Biolog phenotypic tests were employed, along with Matrix Assisted Laser Desorption/Ionization Time-of-Flight Mass Spectrometry (MALDI-ToF MS). Fresh microbial colonies of 24 h for bacteria and 120 h for fungi previously grown on agar media were used. The metabolic patterns were determined with the Biolog System (Biolog, Hayward, CA, USA). In brief, culture suspensions were inoculated in a final volume of 100 µL into GEN III microplates in the case of bacteria and FF microplates in the case of fungi and incubated for 24 h at 33 °C and 26 °C, respectively. The plates were read using the Biolog MicroStation^TM^ 3.5 software, and the results were compared with the database for identification. Metabolic reactions were observed through the reduction of tetrazolium redox dyes, which indicate the utilization of carbon sources and are displayed in various tones of pink and purple [43].

Results obtained using the Biolog Microstation were confirmed by MALDI-ToF MS (VITEK MS PRIME, bioMérieux, Marcy-l′Étoile, France). Microbial biomass obtained from fresh cultures was embedded in a matrix solution. The method involves the thermal desorption of ribosomal proteins from the crystallized matrix under the action of a laser beam, followed by the acceleration of electrically charged particles in vacuum conditions. The time taken by the charged particles to reach the detector differs based on mass and electric charge, thus offering a distinctive spectrum that leads to species-level identification [44].

### 2.5. Metal Tolerance Assessment and Effect on Microbial Growth

The resistance of the isolated microbial strains to chromium, lead, and zinc was characterized based on the minimum inhibitory concentration (MIC) and their tolerance index, as well as through observations on the effect of high concentrations of the mentioned metals on their growth rate.

**Figure 1:**
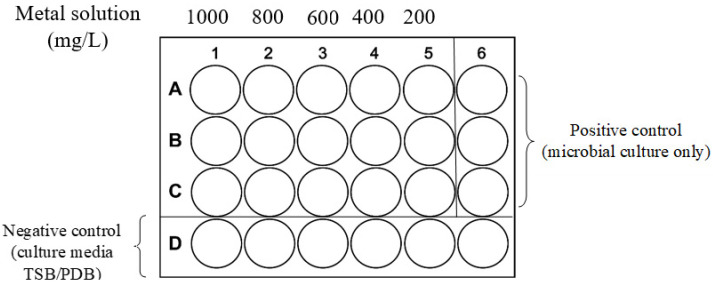
Schematic representation of the MIC protocol in a 24-well plate.

The MIC was determined in 24-well plates (Figure 1) with liquid media supplemented with concentrations between 200 and 1000 mg/L of K_2_Cr_2_O_7_, ZnSO_4_, and Pb (NO_3_)_2_. The culture media used were TSB for bacteria and PDB for fungi. Briefly, a volume of 900 μL medium was inoculated aseptically with 100 μL microbial inoculum from fresh colonies (1 × 10^8^ UFC/mL for bacteria and 1 × 10^7^ UFC/mL for fungi). For bacterial samples, the plates were incubated for 24 h at 35 °C, followed by reading the optical density (OD) at a wavelength of 600 nm using a plate reader. For fungal samples, plates were incubated for 72 h at 28 °C, and growth was observed macroscopically. The MIC was determined as the lowest concentration producing visible growth inhibition or morphological modifications.

The tolerance index to Cr, Pb, and Zn of the isolated strains was determined by two different methods dependent on the type of microorganism, as proposed by Oladipo et al. [45,46] and presented below.

Stock solutions of chromium, lead, and zinc were prepared by dissolving metallic salts in ultrapure water, further sterilized by filtration, to reach a final concentration of 1000 mg/L. Sterile Luria–Bertani Broth (g/L: 10, tryptone; 5, yeast extract; 5 NaCl) was amended with each metallic stock solution and inoculated with 10% fresh bacterial inoculum of OD 1.0. Simultaneously, an inoculated medium without metal served as a positive control, and an uninoculated medium with metal served as a negative control. Samples were incubated for 24 h at 35 °C, and bacterial growth was assessed by reading the optical density at 600 nm at 4 h intervals using the microplate reader. The optical densities of the samples were compared with the control following the incubation period.

For fungi, mycelia disks of 5 mm diameter from colonies pre-grown on PDA for 7 days were inoculated onto Sabouraud agar plates amended with the stock solutions in order to reach a final concentration of 1000 mg/L of K_2_Cr_2_O_7_, ZnSO_4_, and Pb (NO_3_)_2_. Positive and negative controls were also assembled. Plates were incubated at 28 °C for 7 days, and radial growth was measured daily. At the end of the incubation period, the samples were compared with the control. The tolerance index and percentage of inhibition were calculated based on the following equations:

Tolerance Index_Bacteria_ = DO_Me_/DO (1)

where DO_Me_ = Optical density of the sample in the presence of heavy metal and DO = Optical density of the sample without heavy metal.

Tolerance Index_Fungi_ = RG_Me_/RG (2)

where RG_Me_ = Radial growth (mm) of sample in the presence of heavy metal, and RG = Radial growth (mm) of sample without heavy metal. 

Tolerance to the tested heavy metals was rated as follows: 0.00–0.39 (very low tolerance), 0.40–0.59 (low tolerance), 0.60–0.79 (moderate tolerance), 0.80–0.99 (high tolerance), and ≥1.00 (very high tolerance). 

The growth inhibition induced by the contact with the tested heavy metals was calculated based on the equation used by Yaghoubian et al. [47], where the percentage of inhibition is determined by the radial growth (mm) or optical density of the control (C) and the radial growth (mm) or optical density of the sample grown in the presence of heavy metal (T).

Growth inhibition (%) = [(C − T)/C] × 100(3)

### 2.6. Removal Efficiency Assessment

Following the tolerance screening, one multi-metal-tolerant strain displaying tolerance above the moderate level to chromium, lead, and zinc was selected from each group, and their metal removal efficiency was tested according to the methodology applied by Khan et al. [48]. The culture media used for the bioremediation experiments were Luria-Bertani (pH 6.0) for the bacterial strain and PDB (pH 7.0) for the fungal strain, amended with 100 mg/L K_2_Cr_2_O_7_, ZnSO_4_, and Pb(NO_3_)_2_ individually. A volume of 500 µL of fresh inoculum was added aseptically to 50 mL of culture media. Flasks were incubated for 72 h at 37 °C for bacteria and 120 h at 28 °C for fungi in a rotary shaker incubator at 160 rpm. After the incubation period, the samples were centrifuged at 8500 rpm/7350 rcf for 30 min at 4 °C. The biomass was further washed three times with sterile distilled water, dried for 12 h at 60 °C, and weighted. 

The obtained supernatant was used for electrochemical determination of the heavy metal content by using different commercial screen-printed electrodes modified with different nanomaterials.

The metal removal efficiency was determined based on the equation proposed by Emenike et al. [49], where C_0(x)_ is the initial concentration of the metal and C_f(0)_ is the final concentration of the metal:

Heavy metal removal efficiency (%) = [(C_0(x)_ − C_f(0)_)/C_0(x)_] × 100 (4)

### 2.7. Electrochemical Detection of Heavy Metal Concentration in Solution

#### 2.7.1. Electrochemical Measurements

Electrochemical measurements were carried out with a portable miniaturized µStat 4000 Multi Potentiostat/Galvanostat. A conventional electrochemical cell with three electrodes was used, and it consisted of commercial screen-printed electrodes (SPE) on ceramic supports. The working (4 mm diameter) electrodes were made of carbon (SPE, DRP-110) and, respectively, gold (AuSPE, DRP-220AT); the reference electrode was a silver pseudo-reference electrode, and the counter electrode was made of carbon. The electrochemical techniques used in the present study were cyclic voltammetry (CV) and amperometry, respectively. The CV studies were carried out in stationary solutions, using a scan rate of 0.1 V/s, by cycling the potential in different ranges depending on the studied metal. The amperometric studies were carried out in stirred solutions, with all potentials being referred to the Ag pseudo-reference electrode. All experiments were performed at room temperature.

#### 2.7.2. Preparation of the Nanomaterials Based Electrochemical Sensors

The modification of the screen-printed electrodes was performed by using different nanocomposite materials, depending on the desired metal detection, in order to achieve a specific and selective determination of the targeted heavy metal. For chromium determination, the preparation of the sensors was adapted from a method described by Xing et al. [50]. Thus, carbon-paste SPE electrodes were modified with the redox mediator Prussian Blue by direct precipitation of a mixture of 0.1 M K_3_Fe(CN)_6_ and 0.1 M FeCl_3_ in a volumetric ratio of 1:1 onto the working electrode surface. The sensors, denoted as PB/SPEs were kept at room temperature for 10 min and then dried at 65 °C for 3 h. 

For lead detection, carbon-paste SPE electrodes were modified with metallic nanoparticles. Gold nanoparticles (AuNP) with a diameter between 35 and 170 nm were synthesized according to a protocol adapted from Wu et al. [51] and Tukur et al. [52] using tetrachloroauric(III) acid trihydrate (HAuCl_4_·3H_2_O) and sodium citrate. A 0.50 mM HAuCl_4_·3H_2_O solution was stirred at 950 rpm and heated at 98 °C. A pre-heated 0.17 M sodium citrate solution was added, and the stirring and heating were continued for 20 min. The resulting solution was then cooled to room temperature, and a red-violet AuNP dispersion was obtained, which was centrifuged, and the AuNPs were collected and redispersed in ultrapure water.

Synthesized gold nanoparticles (AuNPs) were mixed with a solution of 0.5% chitosan in acetic acid at 2%, and a final volume of 10 μL was drop-casted on the surfaces of the working electrodes. The modified AuNPs-CS/SPEs sensors were maintained for 10 min at room temperature, and then they were dried for 1 h at 65 °C. 

The MWCNTs-CS/PB/AuSPEs-based sensors were used for zinc determination, according to a work published by Ringgit et al. [53]. Thus, in a first step, commercial gold SPE electrodes were modified by drop casting a mixture of chitosan and MWCNTs (1:2) in acetic acid at 1%, followed by drying at room temperature for 2 h. Afterwards, the deposition of Prussian Blue redox mediator was carried out by direct precipitation of an equimolar mixture of 5 mM FeCl_3_ and K_3_Fe(CN)_6_ and drying the obtained sensors for 10 min at room temperature. 

When not in use, all modified electrodes were stored at room temperature in the dark. 

### 2.8. SEM-EDX and FTIR Analysis

The fungal biomass treated with 100 mg L^−1^ K_2_Cr_2_O_7_, ZnSO_4_, and Pb(NO_3_)_2_, respectively, along with the control biomass, grown in unamended media, were subjected to Scanning Electron Microscopy (SEM) (Hitachi, Chiyoda City, Tokio, Japan) coupled with Electron Dispersive X-ray (EDX) (Hitachi, Chiyoda City, Tokio, Japan) analysis to characterize the surface morphology of the samples upon exposure to the metallic ions. For this purpose, the samples were prepared by washing the fungal biomass three times with sterile distilled water, followed by drying it at 60 °C for 8 h. The dried biomass was further weighted and deposited onto carbon tapes, then sputtered (Quorum 150 R ES Plus Sputter Coater) (Quantum Design GmbH, Breitwieserweg, Germany) with a layer of 5 nm of gold under vacuum conditions for improved electrical conductivity. The samples were analyzed using a FEI Quanta 200 scanning electron microscope (FEI Company, Hillsboro, USA) with an acceleration voltage of 30 KV and 133 Pa chamber pressure. The EDX analysis was carried out using a Hitachi TM4000Plus microscope (Hitachi, Chiyoda City, Tokio, Japan).

In order to observe the functional groups involved in the adsorption of the metallic ions, spectral characterization of the treated and untreated biomass was carried out by Fourier-transform infrared spectroscopy (FTIR) using a Perkin Elmer FTIR at a scanning spectrum range between 400 cm^−1^ and 4000 cm^−1^.

### 2.9. Data Analysis

Each experiment was carried out in three replicates, and the mean values of the results along with the standard error were calculated. Tukey’s post hoc test (*p*-value < 0.05) was conducted where considered necessary. The significance threshold was set at a 95% confidence interval and a respective *p* value < 0.05. Correlations between selected parameters were also employed. All statistical analyses were performed using Past version 4.14 and Microsoft Excel (Professional Plus 2019).

## 3. Results and Discussion 

### 3.1. Elemental Analysis of Contaminated Soil

The total concentration of the three heavy metals present in the soil samples is included in Table 1. Values varied among the four soil samples but were generally below the legal limits imposed by the European Union regulations of 200 mg/kg for Cr and Pb and 250 mg/kg for Zn, respectively [54]. The exception was observed for one sample whose concentration of Pb exceeded the legal concentration by 5%, as well as one sample for the concentration of Zn, where the concentration exceeded the legal limit by approximately 47%. As observed in Table 1, the total Cr concentration ranged between 80 and 170 mg/kg, the Pb content ranged between <4 and 211 mg/kg, and the Zn concentration ranged between 108 and 403 mg/kg. In two of the soil samples, the concentration of Pb was below the detection limit. It was observed that the sampling site furthest from the facility, namely 545 F—D4, contained the lowest concentrations of Cr, Pb, and Zn compared to the rest of the samples, whereas for the other three sampling sites, the three heavy metals were detected in similar concentrations. Zn was generally present in higher concentrations compared with Cr and Pb in all four soil samples.

### 3.2. Isolation and Identification of the Metal-Tolerant Microorganisms

**Figure 2:**
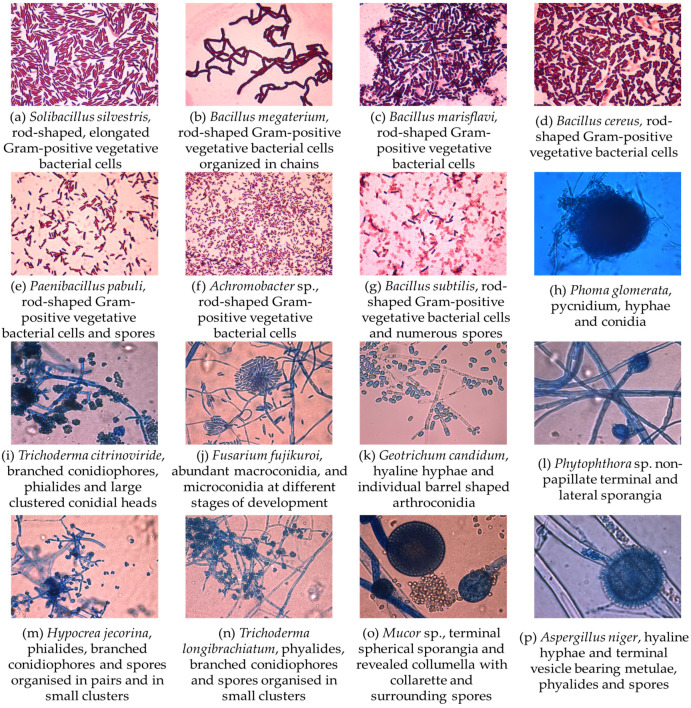
Morphological properties of bacterial isolates: (**a**–**g**)—microscopical appearance; Gram-stain; ob. 100×; respectively; fungal isolates: (**h**–**p**)—microscopical appearance; Lactophenol Cotton Blue stain; ob. 40×.

A number of 16 microorganisms were isolated from the contaminated soil, of which seven strains were Gram-positive bacteria and nine strains were filamentous fungi. Optical microscopy images presenting their morphology are included in Figure 2. The bacteria group consists preponderately of strains from the phyla *Firmicutes* (86%) and *Proteobacteria* (24%). More than 50% of the bacterial strains belong to the *Bacillus* genera, which are frequently found in heavy metal-contaminated soils. The genetic and metabolic diversity ensure the involvement of *Bacillus* spp. in numerous ecological functions, which, along with their various resistant mechanisms against xenobiotics, make them important biotechnological agents [55]. The other species identified, including *Solibacillus silvestris*, *Paenibacillus pabuli*, and *Achromobacter* sp., are often isolated from various environments and are involved in numerous ecological functions such as nitrogen fixation, plant growth, and the production of industrially valuable enzymes [56,57], as well as bioremediation [58].

In regards to fungal species, 77% of the isolated strains belonged to the phylum *Ascomycota*, one strain to the phylum *Zygomycota*, and one to the phylum *Oomycota*. Strains from the genera *Trichoderma*, *Fusarium*, *Mucor*, and *Aspergillus* have been isolated from the contaminated soil. Other studies have also regularly isolated and identified these fungal genera from heavy metal-contaminated environments that also proved to be tolerant to high concentrations of these xenobiotic compounds [59,60,61,62]. Fungi are a highly diverse group, involved in numerous processes such as biological control, bioremediation, degradation of organic materials, and the sequestration and cycling of nutrients along the local trophic web [63]. Contamination with heavy metals and other xenobiotics decreases microbial diversity and relative abundance, more significantly for bacteria compared to fungi, leading to the changing of the community structure based on the “survival of the fittest” principle [64,65,66,67,68].

### 3.3. Metal Tolerance Assessment and Effect on Microbial Growth

The minimum inhibitory concentration (MIC) is presented in Table 2. For the isolated bacterial strains, Pb proved higher toxicity, displaying growth inhibition at the lowest concentration tested of 200 mg/L for 85% of the strains, compared to 57% for Cr and Zn, respectively. Two strains, specifically *B. megaterium* and *B. cereus*, were equally sensitive to the three metals, with growth inhibition beginning below 200 mg/L. The highest MIC observed was 800 mg/L in the case of *S. silvestris* in the presence of Pb, *B. marisflavi* in the presence of Cr, and *B. subtilis* in the presence of Zn. The strain *Achromobacter* sp. was moderately tolerant to Cr and Zn, with the MIC being 600 mg/L in both cases. *P. pabuli* was more tolerant to Zn compared to Cr and Pb, with the MIC observed in that case being 400 mg/L. Our findings correlate with the results confirmed by Altuğ et al. [69], who observed a higher resistance to Cr compared to Pb and Zn, with the MIC value being >2.5 mM for *Bacillus* sp. and *Pseudomonas* sp. Comparatively, fungi displayed higher tolerance in the sense that the growth of the majority of strains was inhibited at concentrations above 400 mg/L of metal salts, with the exception of *P. glomerata*, for which the MIC was 200 mg/L in the case of Cr. For 50% of the fungal strains, the highest MIC was 1000 mg/L in the presence of Zn, higher than the value observed for bacteria. *H. jecorina* was the only strain that presented a MIC of 1000 mg/L for both Pb and Zn. A total of 30% of the strains displayed a MIC of 800 mg/L in the presence of Cr and 20% in the presence of Zn. One strain, *T. longibrachiatum*, was evenly tolerant to the three metals, with the MIC observed being 800 mg/L. 

Fungal strains appeared to be more tolerant to Zn, followed by Cr and Pb, whereas bacteria were more tolerant to Cr and Zn. Similar results were obtained by Rajapaksha [70] and Zeng et al. [71], who concluded that fungi were less sensitive to high concentrations of heavy metals compared to bacteria. Furthermore, the toxicity of Zn and Pb has been observed by Perelomov et al. [72] for Gram-positive as well as Gram-negative bacteria. In their study, observations were carried out for environmentally relevant species *Pseudomonas chlororaphis*, *P. fluorescens*, and *Phodococsus* RS67. In the case of fungi, Cr and Pb have been proven to be highly toxic. Muñoz et al. [73] observed that the MIC values were higher in the presence of Zn compared to Pb for fungal strains such as *Penicillium* sp., *Galactomyces geotrichum*, *Pseudallescheria boydii*, and *Trichosporon* sp. MIC values were similar for Cr and Pb.

**Figure 3:**
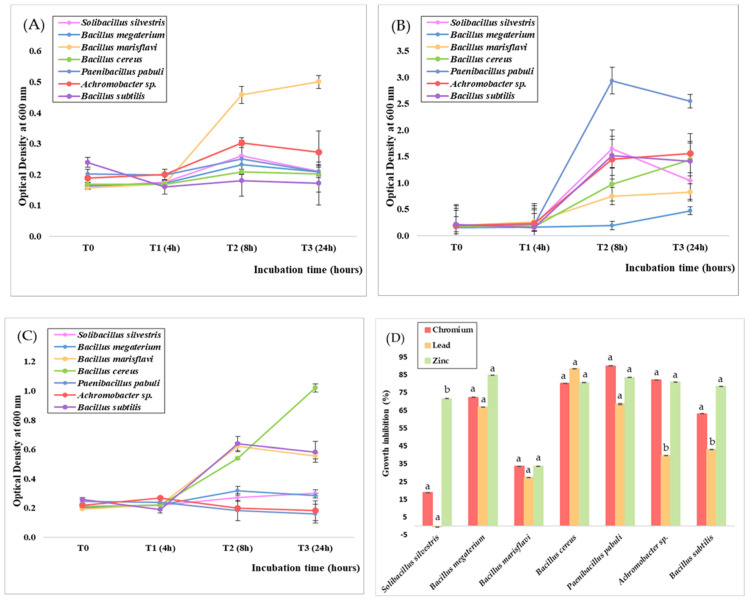
Growth curves of bacterial isolates in Luria-Bertani medium supplemented with 1000 mg/L K_2_Cr_2_O_7_ (**A**), Pb (NO_3_)_2_ (**B**), and ZnSO_4_ (**C**) and growth inhibition (**D**). Different letters indicate statistically significant differences between values.

To assess the effect of Cr, Pb, and Zn on microbial growth, the differences observed over a span of 24 h on the seven bacterial strains are highlighted in Figure 3. Zn was proven to exhibit a significantly higher bacteriostatic effect, inhibiting more than 50% of the growth of 85% of the strains, followed by Cr, with an efficiency of inhibiting bacterial growth in more than 70% of the strains, whereas Pb exhibited the least bacteriostatic effect, reducing the growth rate in about 42% of the strains. 

The antibacterial efficiency of Zn ions observed through our research is consistent with previous studies. The study conducted by Mwandira et al. [74] concluded that concentrations of 10 mg/L and 50 mg/L of ZnCl_2_ completely inhibited bacterial growth, compared to Pb, as values above the threshold level determine the displacement of essential metals. Danilova et al. [75] observed that even low concentrations (0.005 M) of Zn in the form of ZnSO_4_ inhibited the growth of *S. pyogenes* and *E. coli* as well as biofilm formation. Abdalkader and Al-Saedi [76] tested the antibacterial efficiency of ZnSO_4_ in low concentrations, between 2 and 20 mg/mL, on a series of multidrug-resistant pathogens, such as *S. aureus*, *S. epidermis*, *Proteus* spp., *P. aeruginosa*, *Enterobacter* spp., and *Klebsiella* spp. In soil, long-term application of Zn salts affects the structure of the bacterial community in terms of species abundance [77]. Zn has also been repeatedly studied, particularly in the composition of nano-scale products, with antibacterial efficiency against Gram-positive and Gram-negative bacteria, including *S. aureus*, *E. faecalis*, *E. coli*, and *S. typhimurium* [78]. 

Similar results were observed in the presence of Cr, with the growth of 71% of the strains being inhibited above 50%, proving its bacteriostatic effect. On the other hand, Pb inhibited more than 50% of the growth of only 42% of the strains. An exceptional case was observed in the case of *S. silvestris*, for which the presence of a high concentration of Pb (NO_3_)_2_ stimulated the growth by 0.5%. Also, *B. marisflavi* was the only strain for which less than 35% of its growth was inhibited in the presence of each of the three metal salts. The higher toxicity of Cr compared to Pb can be explained by the uptake particularities employed by bacterial cells. Cr, in the form of the Cr^6+^ ion, is highly soluble and easily passes through sulfate transport channels due to its structural similarity with the SO_4_^2−^ anion [79]. Pb is bound inside the cell as Pb^2+^ by metallothioneins that are synthesized as a response to the presence of metals in the environment [80]. Both processes require energy expenditure, but Cr could be more readily available due to its competition with sulfur, which is an essential nutrient required for growth and normal metabolic functioning [81]. 

**Figure 4:**
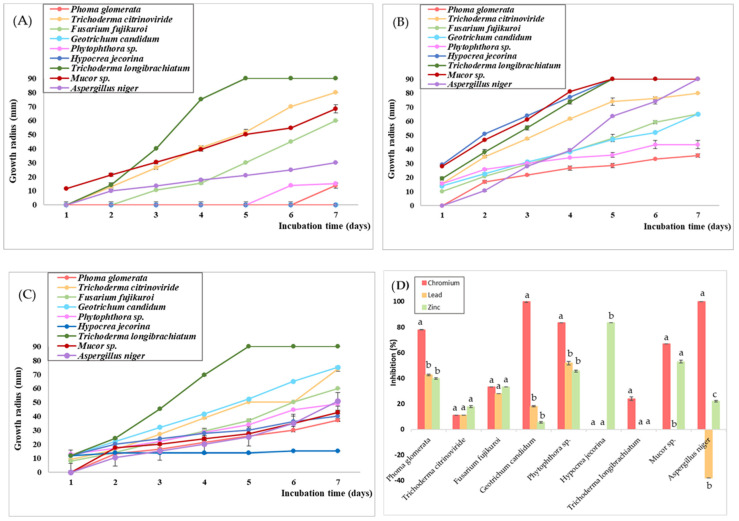
Growth curves of fungal isolates in PDB medium supplemented with 1000 mg/L K_2_Cr_2_O_7_ (**A**), Pb (NO_3_)_2_ (**B**), and ZnSO_4_ (**C**) and growth inhibition (**D**). Different letters indicate statistically significant differences between values.

In regards to the fungistatic effect of the highest concentrations of K_2_Cr_2_O_7_, Pb(NO_3_)_2_, and ZnSO_4_ tested (Figure 4), it was observed that Cr exerted the highest toxicity, inhibiting more than 50% of the growth of 33% of the strains. 

The high concentration of K_2_Cr_2_O_7_ also exerted a complete fungicidal effect in the case of two strains, namely *G. candidum* and *A. niger*. On the other hand, for one strain, namely *H. jecorina*, no inhibition of growth was observed. In the case of Zn, the presence of Zn^2+^ ions had a moderately inhibitory effect, stagnating more than 50% of the growth only for two strains, *Mucor* sp. and *H. jecorina*. The inhibition of mycelial growth was more significant for the latter, with the growth rate being reduced by 83%. One strain, *T. longibrachiatum*, did not exhibit any inhibition in mycelial growth. Contrastingly, Pb exhibited a significantly lower fungistatic effect, with no growth inhibition observed in 33% of the strains. An important mention is for *A. niger*, whose growth was stimulated by almost 40%. Similar results were obtained by Prakash et al. [82], who observed a positive correlation between increasing Pb concentrations and mycelial growth in media containing up to 200 ppm Pb, stimulating maximum growth. 

Pb tolerance is generally sustained by increased proline synthesis in the presence of Pb^2+^, which acts as a detoxifying mechanism, an antioxidant defense mechanism, and a signaling agent [83]. Our results are consistent with the findings of Tian et al. [84], who observed that concentrations below 1000 mg/L stimulated the bioactivity of *A. niger*, represented by increased respiration and production of antioxidants. Long et al. [85] also observed that concentrations up to 300 mg/L of Pb(NO_3_)_2_ determined an increase in sclerotial biomass as a cause of the oxidative stress induced by the presence of Pb^2+^ ions. 

A low dose of toxic compounds induces a hormesis effect that determines long-term adaptation to environmental stressors [86]. Various heavy metals have been proven to pose a stimulating effect on mycelial growth in low concentrations. For instance, a concentration of 3 ppm of Pb was able to stimulate the growth of *Pythium debaryanum* [87]. Other heavy metals, such as copper [88], cobalt [89], and cadmium [90], were proven to stimulate mycelial growth in low concentrations as a result of an increase in metabolic activity employed to withstand stressful conditions. Our findings at a much higher concentration of lead indicate the potential of *A. niger* for future bioremediation studies.

**Figure 5:**
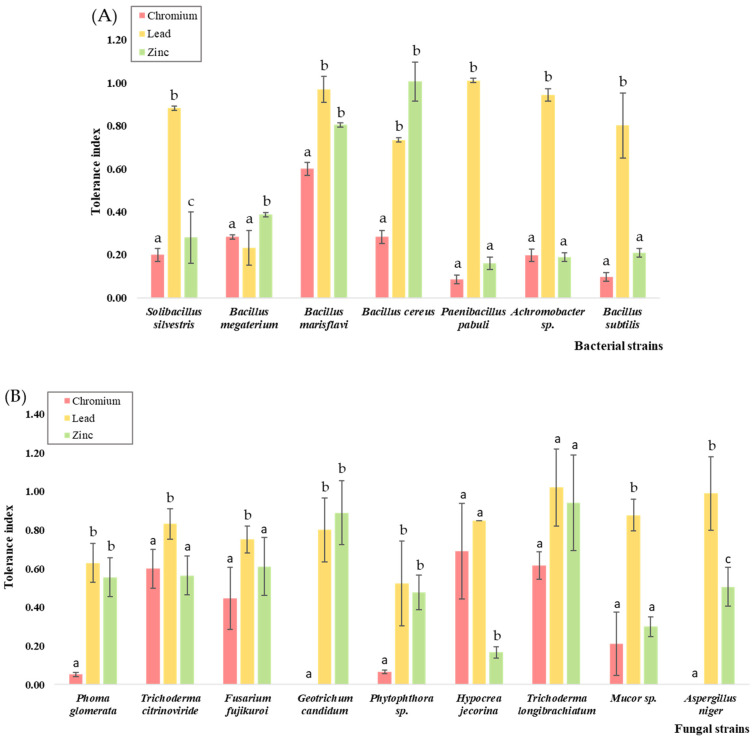
Tolerance index of the (**A**) bacterial strains and (**B**) fungal strains to the concentration of 1000 mg L^−1^ of K_2_Cr_2_O_7_, Pb(NO_3_)_2_, and ZnSO_4_. Different letters indicate statistically significant differences between values.

The heavy metal resistance of the microbial strains was tested at a concentration of 1000 mg/L of K_2_Cr_2_O_7_, ZnSO_4_, and Pb(NO_3_)_2_. The tolerance index ranged between 0.0, indicating a very low tolerance, and 1.02, indicating a very high tolerance (Figure 5).

Compared to the bacterial strains, the fungal strains were more tolerant to the three metals tested, which aligns with previous findings. The genera *Trichoderma* sp. showed generally high tolerance to Cr, Pb, and Zn. Out of the 9 fungal strains, *T. longibrachiatum* exhibited no growth reduction in the presence of Pb and Zn and a very low reduction of 24% in growth in the presence of Cr compared to the other strains, indicating its high tolerance to the three metals. Similar studies support our observations, with various *Trichoderma* isolates being resistant to high concentrations of heavy metals such as Ni, Cd [91], As, Pb [92], Co, Hg, and Zn [93].

Out of the three metals tested, Cr exhibited the highest level of toxicity towards both bacteria and fungi, as observed by the generally low tolerance values as well as the complete inhibition manifested in the cases of *G. candidum* and *A. niger.* The highest TI values observed for the microbial strains in the presence of Cr varied between 0.60 and 0.69. Moderate tolerance was observed in one bacterial strain, namely *B. marisflavi*, and in three fungal strains, *T. citrinoviride*, *T. longibrachiatum*, and *H. jecorina*, displaying moderate tolerance. According to Zapana-Huarache et al. [94], *Trichoderma* sp. presented higher tolerance to concentrations up to 1000 mg/L of K_2_Cr_2_O_7_ compared to other fungal strains such as *Penicillium* sp., yet still maintained a much lower level of tolerance compared to our results, with a TI of ≈0.2. Similar results were obtained by Kumar & Dwivedi [95], who observed a very low tolerance of approximately the same value for *Trichoderma* spp. In the presence of 1000 mg/L of K_2_Cr_2_O_7_ or even complete growth inhibition. Such an effect is caused by the accumulation of ROS, which induces significant cellular damage.

A similar sensitivity was observed in the presence of Zn, with only 37% of the strains displaying tolerance above the moderate level. The lowest value above the moderate limit was observed for *F. fujikuroi*, with the TI being 0.61. The other strains, including *B. marisflavi* and *B. cereus* for the bacteria group and *G. candidum* and *T. longibrachiatum* for the fungi group, presented high tolerance to zinc; respectively, *B. cereus* exhibited a very high tolerance with a TI > 1. Higher concentrations of Zn are known to hinder microbial growth due to its strongly oxidative nature [96]. Out of the three metals tested, 87% of the microbial strains displayed tolerance above the moderate level for Pb, with the lowest TI value registered being 0.63 in the case of *P. glomerata.* For bacteria, 57% of the strains displayed high tolerance, with TI values between 0.80 and 0.97, and one strain, *P. pabuli*, displayed a TI value of 1.01, indicating a very high tolerance. In regards to Pb toxicity for fungi, 55% of the strains displayed high tolerance, with TI values between 0.80 and 0.99. The strain *T. longibrachiatum* registered a TI of 1.02, having the highest tolerance to Pb out of the microbial strains studied.

Our results indicate a higher tolerance to Pb, followed by Zn, and a moderate tolerance to Cr for both bacteria and fungi. In contrast, Campillo-Cora et al. [97] reported a higher tolerance of the bacterial community to Zn compared to Pb, whereas Bérard et al. [98] proved that long-term exposure to Pb, even in a polymetallic contamination context, along with Zn, Cu, or Cd, induced Pb tolerance in both bacteria and fungi, inhibition being less intense in fungal communities. Compared to Cr, Zn, Co, Cd, Ni, and Cu, Pb tolerance was higher in bacteria at concentrations up to 2000 mg/L, which is attributed to the role of proline in lessening the damage produced to membranes and proteins attributed to increased oxidative stress [99]. The tolerance of the microbial community to heavy metals in soil is positively dependent on soluble or bioavailable metal fractions as well as soil parameters such as soil organic matter and pH [100]. Thus, long-term contamination with heavy metals determines an increase in metal tolerance as well as the dominance of resistant species.

From each group, one strain displayed multi-metal tolerance above the moderate level for Cr, Pb, and Zn, namely *B. marisflavi* and *T. longibrachiatum.* These strains proved to be moderately tolerant to Cr, with similar TI values of 0.60 and 0.62, respectively, whereas both strains displayed high and very high tolerance to Zn, followed by Pb, as observed by their TI values. We observe that *T. longibrachiatum* was overall more tolerant to the three metals compared to *B. marisflavi*, with the TI for Zn being 0.94 and 1.02 for Pb, whereas in the case of *B. marisflavi*, the values were slightly lower: 0.81 for Zn and 0.97 for Pb. 

The metal tolerance of both genera has been extensively studied for bioremediation applications. Prakash et al. [82] assessed the tolerance of *Trichoderma* spp. to increasing concentrations of Pb and Zn. Low concentrations of Pb, up to 100 ppm, stimulate mycelia growth, with a decreasing tendency dependent on increasing Pb concentration. On the other hand, *Trichoderma* spp. proved to be more sensitive to increasing concentrations of Zn. Tansengco et al. [101] confirmed the tolerance of various *Trichoderma* isolates, such as *T. virens*, *T. harzianum*, *T. gamsii*, and *T. saturnisporum*, to high concentrations of Cr, Pb, Zn, Cu, and Ni, up to 1000 ppm.

For the *Bacillus* genera, several studies indicate the bioremediation potential of various *Bacillus* sp. isolates. Mardiyono et al. [102] observed the efficiency of *Bacillus subtilis* for the bioremediation of Ni sourced from the electroplating industry. Two novel *Bacillus* sp. strains were proven to be tolerant to concentrations of 100 mg/L Pb and Cd, with bioremediation efficiency using biosorption mechanisms, in the study conducted by Heidari and Panico [103]. Guo et al. [104] observed a 95% reduction in the concentration of Cr(VI) using *Bacillus megatherium* through enzymatic reduction, biosorption, and precipitation with phosphate. *Bacillus altitudinis* was observed to exhibit high tolerance to concentrations up to 20 mM Zn, as well as Cu, Ni, Cr, Pb, and Hg [105]. Further, Arroyo-Herrera et al. [106] reported the multi-metal resistance of *Bacillus* sp. to Co, Cr, Cu, Ni, Zn, and As. 

Ultimately, the present study provides valuable results regarding the use of both *B. marisflavi* and *T. longibrachiatum* strains for the efficient removal of heavy metals.

### 3.4. Removal Efficiency Assessment

**Figure 6:**
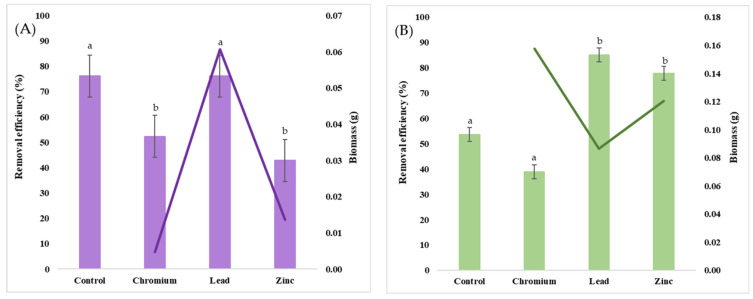
Biomass dry weight (g) (clustered columns) correlated with removal efficiency (%) (purple and green lines) of *Bacillus marisflavi* (**A**) and *Trichoderma longibrachiatum* (**B**). Different letters indicate statistically significant differences between values.

The bioremediation assay was carried out at a concentration of 100 mg L^−1^ K_2_Cr_2_O_7_, Pb(NO_3_)_2_, and ZnSO_4_ individually, and the removal efficiency was quantified by detecting the differences in the concentration of the three metal salts prior to and after the microbial treatment using modified electrochemical sensors. Figure 6 depicts the removal efficiency of the two selected strains in correlation with the biomass produced during the incubation period in the presence of K_2_Cr_2_O_7_, Pb(NO_3_)_2_, and ZnSO_4_.

It was observed that *T. longibrachiatum* was significantly more efficient in removing Cr and Zn from solution, decreasing more than 45% of their concentration compared to *B. marisflavi*. Out of the three metals tested, *T. longibrachiatum* showed the highest removal efficiency in the case of Cr^6+^, decreasing 87% of its concentration in solution. The observed percentage removal of Zn^2+^ from solution was 67% and 48% for Pb^2+^, respectively. Interestingly, the presence of Cr inhibited biomass growth, from 0.10 g of the control to 0.7 g, whereas in the presence of Pb and Zn, biomass production was stimulated, increasing to 0.15 g and 0.14 g, respectively. Morphological alterations were also observed for the biomass grown in media amended with Cr, Pb, and Zn, respectively, characterized by the clumping and breakage of the hyphae as well as elongations and deformations. A negative correlation was observed between biomass production and removal efficiency, as the lowest biomass produced bioaccumulated the highest percentage of metal ions. The high concentrations of heavy metals exert severe stress on the fungal strain, inhibiting growth as metabolic mechanisms are employed for controlling the accumulation of ROS as a result of oxidative stress [107]. The bioaccumulation of metal ions from the environment involves passive processes such as biosorption onto the cellular surface as well as active processes, namely intracellular sequestration [108]. Biosorption is one of the first mechanisms employed in contact with pollutants, involving ion exchange, complexation, and physical adsorption due to the negative charge of the functional groups within the structure of the cell wall [109]. 

The uptake efficiency of the biomass of *B. marisflavi* was the lowest in the case of Cr, with a decrease in concentration of about 7%, followed by Zn, with a 19% decrease in concentration. On the other hand, a reduction of 87% in the concentration of Pb was obtained. Visible morphological modifications were also observed in the Pb-treated samples, with the biomass acquiring a brown color, attributed to the biotransformation of Pb^2+^ ions through precipitation catalyzed by phosphatase [110,111]. The high tolerance of the bacterial strain to Pb was positively correlated with its bioremediation efficiency, having the highest tolerance index compared to Zn and Cr as well. Similar results in regards to Pb remediation were obtained by Njoku et al. [112] using *Bacillus megaterium* with an efficiency of removing 73% of Pb from solution, whereas Mohapatra et al. [113] obtained over 97% removal of Pb^2+^ using live and dead *Bacillus xiamenenensis* biomass under optimized conditions. The low removal efficiency of *B. marisflavi*, despite its high tolerance to Cr and Zn, is linked to the particularities of its resistance mechanisms. Shaw & Dussan [114] demonstrated the behavior of efflux pumps in *Bacillus cereus* B6 in the presence of Pb, As, and Cr as a detoxifying mechanism employed in order to control the intracellular metal concentration. Furthermore, post-efflux mechanisms are initiated to restrict the metal ions from being reabsorbed within the cell [115]. Another resistance mechanism involves the precipitation of the metal into insoluble salts for the purpose of reducing their bioavailability [116]. 

Studies on the efficiency of *B. marisflavi* in removing heavy metals from the environment are scarce [117,118]. The strain has been observed to degrade benzyl butyl phthalate and dimethyl phthalate [119], as well as polycyclic aromatic hydrocarbons (PAHs) [120] and pesticides such as chlorpyrifos [121], indicating its vast metabolic capacities for bioremediation. Mishra & Doble [117] proved the efficiency of *B. marisflavi* to reduce more than 90% of the concentration of Cr^6+^ from a solution containing 200 mg/L of K_2_Cr_2_O_7_, but at a lower pH of 4.0. Therefore, additional studies are required to determine the influence of pH on bioremediation efficiency as well as understand the particular resistance mechanisms employed by *B. marisflavi*. Kayalvizhi & Kathiresan [118] obtained a removal of 70% of Zn, 62.5% of Cu and Pb, and 44% of Mn from solution using *B. marisflavi* under optimized conditions. Through our study, we have proved the high efficiency of *B. marisflavi* for Pb bioremediation.

Resistant strains are expected to be able to uptake substantially more metal ions from the environment compared to sensitive strains. Numerous studies show a positive correlation between the high tolerance index of microbial strains and their removal efficiency for various metals [122,123,124,125], yet our findings indicate the absence of such correlation in the case of Zn and Cr, proving the necessity of more in-depth studies on microbial heavy metal resistance as well as removal mechanisms. Microorganisms possess various resistance mechanisms against heavy metals with the primary role of preventing toxicity, which may not consequently determine the removal of heavy metals from the environment, such as extracellular efflux, metal-sensing regulators that repress genes involved in metal uptake, and enzymatic reduction [126]. 

### 3.5. Electrochemical Detection of Heavy Metals Concentration

#### 3.5.1. Cyclic Voltammetry Studies

For the chromium detection using the PB/SPE sensors, the cyclic voltammograms recorded in a solution containing 0.1 M KCl and 0.1 M HCl highlighted the presence of two cathodic peaks at 0.01 V and 0.28 V potentials, which were attributed to the PB and Cr^6+^ reduction, respectively. The electrochemical properties of PB emphasized by the cyclic voltammograms obtained in the electrolyte solution in the presence of a cathodic and an anodic peak are based on the following coupled reactions:

PB ⟺ Everitt’s salt and

PB ⟺ Prussian yellow (PY).

The corresponding equations are:

KFe^III^[Fe^2+^(CN)_6]_ + K^+^+e^−^ ⟺ K_2_Fe^II^[Fe^2+^(CN)_6_] (5)

KFe^III^[Fe^2+^(CN)_6_] ⟺ Fe^III^Fe^3+^(CN)_6_ + K^+^ + e^−^ (6)

As can be seen in Figure S1, an increase in the cathodic peak current with an increase in chromium concentration is observed, demonstrating the electrocatalytic behavior of the redox mediator towards chromium reduction. A value of +0.3 V vs. Ag/AgCl was selected as a working potential for further amperometric detection of Cr^6+^.

The formal reduction potential of Pb^2+^ at the surface of AuNPs-CS/SPE sensors was determined to be −0.45 V vs. Ag/AgCl, and this potential value was further used in the amperometric studies in order to determine the analytical performance of the developed sensor for Pb^2+^ detection (Figure S2).

The determination of Zn^2+^ content was carried out by cyclic voltammetry using the developed MWCNTs-CS/PB/AuSPE based sensors. The voltammograms were recorded in 0.1 M KCl and 0.1 M HCl as supporting electrolyte solutions in the absence and presence of different concentrations of Zn^2+^, from 0.01 to 4.17 mM, by sweeping the potential from −0.5 to −2.0 V vs. Ag/AgCl. (Figure S3).

The intensity of the reduction peaks increases with the increase in zinc concentration, thus indicating the electrocatalytic effect of the developed sensor for the detection of Zn^2+^ due to the increase in the active surface and the kinetic transfer of electrons (Figure S3A). It was also observed that the cathodic potential peak shifted with the increase in Zn^2+^ concentration to lower values. 

The cyclic voltammetry technique was used to obtain the calibration curve for Zn^2+^ by using the developed sensor (Figure S3B). The analytical performances of the developed MWCNT-CS/PB/AuSPE sensor for Zn^2+^ determination were very good, with a specific sensitivity of 3.682 A·M^−1^·cm^−2^ being obtained for a linear concentration range extended up to 0.833 mM and a detection limit of 0.2 μM.

#### 3.5.2. Amperometric Studies

The Cr^6+^ detection was performed by using the PB/SPE-based sensor at an applied working potential of +0.3 V vs. Ag/AgCl by successive additions of increasing volumes of 0.1 M potassium dichromate (Figure S4).

The optimization of the working potential for chromium determination was performed by carrying out calibrations of the sensor at different applied potential values (Table S1). An applied potential value of +0.3 V vs. Ag/AgCl will be used for the determination of chromium content in real samples.

The reproducibility of PB-based sensors for Cr detection was determined by carrying out calibrations with the other three PB/SPE sensors in the same conditions. The obtained average specific sensitivity was 336.8 ± 42.1 mA·M^−1^·cm^−2^ (RSD = 11.6%), demonstrating the good reproducibility of the sensors modified with Prussian Blue redox mediator for the detection of Cr.

The stability studies were carried out by amperometric measurements, with an average current value determined for the PB-based sensor of 8.70 ± 0.96 μA (RSD = 11.6%) being obtained, demonstrating in this way the good inter-operational stability of the PB/SPE-based sensor for Cr determination (Figure S5).

For the amperometric determination of lead, calibration of the AuNP-CS-based sensor was performed at an applied potential value of −0.45 V in the electrolyte solution of 0.1 M Tris-HCl (Figure S6). 

The determination of Pb^2+^ was achieved in a linear range of concentrations from 0.017 to 0.515 mM, with a specific sensitivity of 20.66 mA·M^−1^·cm^−2^ and a detection limit of 34.7 µM. Thus, the developed sensor based on AuNPs-CS showed good analytical performance for Pb detection and was further used for the determination of the heavy metal in the real samples.

### 3.6. Electrochemical Detection of Heavy Metals in Treated and Untreated Supernatant

After the development and electrochemical characterization of the sensors modified with different nanomaterials, they were further used to determine the heavy metal content in control solutions containing uninoculated culture media amended with a known concentration of Cr^6+^, Pb^2+^, and Zn^2+^ (100 mg/L), respectively, and in test solutions represented by culture media amended with metal salts and inoculated with individual microbial strains with potential for heavy metal bioremediation. 

The concentrations of Cr^6+^, Pb^2+^, and Zn^2+^ determined with the nanomaterial-based electrochemical sensors, as well as the decrease in heavy metal content due to microorganism biosorption expressed as a percentage, are shown in Table 3. 

Thus, the determination of Zn^2+^ in real samples was carried out by cyclic voltammetry, while Cr^6+^ and Pb^2+^ detection were achieved by amperometric measurements. The equations used for the calculations are: Y (µA) = 42.898 (mM) X + 5.306 for Cr^6+^; Y (µA) = 2.594 (mM) X + 0.803 for Pb^2+^; and Y (µA) = 462.78 (mM) X + 285.9 for Zn^2+^, respectively. The values of the reduction currents recorded for each injected sample volume were interpolated on the calibration curves previously determined for the sensors used. 

The bioremediation capacity of the microorganisms for each heavy metal species was correlated with the percentage decrease in metal concentration in the culture medium before and after incubation with the bacteria or fungi species. 

The highest decrease in heavy metal concentration was observed for the sample containing Pb^2+^ and inoculated with the bacterial strain *B. marisflavi* and for the sample with Cr^6+^ inoculated with the fungal strain *T. longibrachiatum*, with values of over 85%, which shows a significant bioremediation capacity. 

### 3.7. SEM/EDX Characterization of the Microbial Biomass

The surface morphology of the biomass of *B. marisflavi* and *T. longibrachiatum* before and after treatment with K_2_Cr_2_O_7_, Pb(NO_3_)_2_, and ZnSO_4_ was analyzed by Scanning Electron Microscopy coupled with Energy-Dispersive X-ray spectroscopy (SEM/EDX) to assess the impact of exposure to heavy metals. For *B. marisflavi*, only the biomass treated with Pb(NO_3_)_2_ and ZnSO_4_ was further analyzed, as the Cr-treated sample was detected to have the lowest remediation efficiency, removing less than 10% of the metal ions from solution, and no macroscopic alterations of the biomass were observed.

The SEM micrographs of *B. marisflavi* presented in Figure S7 exhibited normally developed bacterial cells, characterized by numerous rod-shaped bacteria with a smooth surface, entrapped in a biofilm structure for the control sample. As expected, EDX analysis indicated the absence of Pb and Zn. 

Following the adsorption of Pb from solution, deformations and irregularities in the shape of the cells were observed, as well as their aggregation in an unregulated shape with a distinctive metallic glow as a result of Pb^2+^ biosorption [127]. The EDX spectral image indicates the adsorption of 9.5% Pb^2+^ onto the surface of the cell. Furthermore, differences in the atomic percentage of elements oxygen (O) and phosphorous (P) were observed following Pb uptake, represented by a decrease from 24.1% to 8.5% for O and, respectively, an increase from 3.1% to 5.4% for P, indicating the formation of covalent bonds between Pb^2+^ and functional groups containing O and P elements such as amino and phosphate [128]. For the Zn-treated biomass, deformations were observed regarding the shape of the cells, although less severe compared to the Pb-treated biomass. EDX imaging confirms the presence of Zn^2+^ on the surface of the cell in a percentage of 0.4%, as well as differences in the percentage of O and P, similar to the Pb-treated sample. A decrease in oxygen from 24.1% to 18.3% was observed, as well as an increase in P from 3.1% to 4.9%. Also, an increase in K from 2.3% to 5.4% was detected, indicating interactions with the specific functional groups. The low percentage of Zn^2+^ indicates that adsorption is not the primary uptake mechanism of *B. marisflavi*, whereas extracellular uptake is more prominent in contact with Pb. The greater affinity of the bacterial biomass for Pb compared to Zn can be attributed to the larger ionic size and higher electronegativity of the former, an effect observed by Wierzba [129] as well.

Compared with the biosorption efficiency reported by other studies focusing on *Bacillus* sp. [130,131], for which Pb was detected in a percentage of 1.77% and 4.77%, respectively, onto the surface of the cell, the present study indicates a better biosorption potential. On the other hand, other studies obtained a removal of lead through biosorption of 79%, 85%, and 87%, respectively, using *B. licheniformis*, *B. subtilis*, and *B. cereus* [132], thus indicating the diversity of bioremediation mechanisms by different *Bacillus* strains. The peptidoglycan layer of Gram-positive and Gram-negative bacteria determines the anionic nature of the cell wall, allowing for metal binding to its surface. Additionally, teichoic acid and teichuronic acid are included within the structure of the cell wall of Gram-positive bacteria, providing more binding sites compared to Gram-negative bacteria [133]. The biosorption capacity varies as a consequence of the dissimilarities in the functional groups present within the cellular structure of the microorganisms; the main binding sites for metal ions are carbonyl, carboxyl, hydroxyl, amino, and sulfhydryl groups [134].

SEM micrographs of *T. longibrachiatum* biomass presented in Figure S8 highlight the structural alterations exerted by contact with heavy metals. The control sample presents normally developed hyphae, uniform in shape, organized as thin, smooth clusters. Following treatments with 100 mg/L of Cr, Pb, and Zn, visible morphological alterations were observed. Mycelia of *T. longibrachiatum* exhibited elongation, thickening, and clustering of hyphae, as well as dense depositions on the surface, more visible for the Cr and Zn-treated samples, indicating the adsorption of Cr^6+^, Pb^2+^, and Zn^2+^ ions. Such modifications have also been reported in other studies [135,136,137]. The structural alterations are a result of interferences in the deposition of chitin within the cell wall structure as well as the adsorption of metal onto the cell surface, which determine morphological modifications of the hyphae such as shrinkage and wrinkles, as indicated by Tu et al. [138].

EDX spectral images offered supporting results for the premise of metallic ions adsorbed onto the cell surface. In the case of the Cr-treated sample, Cr was detected at an atomic percentage of 20.5% adsorbed onto the surface of the cell. Compared with the control sample, a decrease in the percentage of C from 71.3 to 67.3 was observed, indicating interactions between C-rich functional groups and Cr^6+^ ions. Furthermore, an increase in O, K, and Al from 21.5%, 3.2%, and 0.9% to 34.8%, 4.9%, and 9.2%, respectively, suggests the sequestration of Cr^6+^ within complexes such as aluminum polyphosphates [139]. On the other hand, the presence of Zn^2+^ and Pb^2+^ was detected in much lower amounts, around 0.1%, respectively. A decrease in concentration for C, K, P, Al, and S was detected from 71.3%, 3.2%, 2.2%, 0.9%, and 0.7% to 60.7%, 0.5%, 0.7%, 0.5%, and 0.1% in the case of the Pb-treated biomass, as well as 57.7%, 1.0%, 1.1%, 0.1%, and 0.2%, respectively, for the Zn-treated biomass. Such observations indicate the precipitation of Pb^2+^ and Zn^2+^ ions as insoluble metal complexes on the surface of the cell wall [140].

Thus, it was observed that a significant percentage of Cr was removed by biosorption, whereas Pb and Zn uptake was mediated primarily by intracellular bioaccumulation. Similar findings were reported by Zhang et al. [141] for *T. brecivompactum* as well as Hlihor et al. [142] for *T. viride*, although *T. longibrachiatum* presented a stronger biosorption efficiency compared to the latter, adsorbing 20.5% Cr as opposed to 3.1%. Also, the *T. longibrachiatum* strain used in the present study presented higher Cr biosorption compared to other genera that have been the subject of recent studies, such as *Aspergillus terriocola* [143] and *Penicillium simplicissimum* [144].

### 3.8. FTIR Characterization of the Microbial Biomass

Figure S9 presents the FTIR spectra of *B. marisflavi* biomass in the absence and presence of Pb and Zn, respectively. The presence of Pb^2+^ and Zn^2+^ ions induced changes in the peaks of the functional groups. For the Pb-treated sample, the intensity of the broad peak observed at 3279 cm^−1^ ascribed to the O-H stretching vibration decreased slightly to 3278 cm^−1^. A more significant difference is observed by the appearance of two peaks at 2874 cm^−1^ and 2854 cm^−1^, attributed to the symmetric and asymmetric C-H stretching vibrations. A similar effect was observed in the case of the Zn-treated sample, characterized by the appearance of a band at 2849 cm^−1^, indicating the interaction of the metal ions and the alkene group [145]. The peaks at 1635 cm^−1^ and 1538 cm^−1^ ascribed to the C=O stretching vibrations of the amide I and amide II groups did not suffer significant modifications, with a slight increase at 1636 cm^−1^ in the case of the Zn-treated sample and a slight decrease to 1537 cm^−1^ for the Pb-treated sample. The peaks in the range 1450–1150 cm^−1^ are attributed to the carboxyl and phosphate groups, where notable differences are observed for the Zn and Pb-treated samples. A decrease in the peak at 1393 cm^−1^ to 1390 cm^−1^ and 1380 cm^−1^, respectively, as well as an increase in the intensity of the peak from 1227 cm^−1^ to 1232 cm^−1^ were detected. Furthermore, the appearance of new peaks at 1310 cm^−1^ and 1155 cm^−1^ suggests the interaction of the Zn^2+^ and Pb^2+^ ions with the aforementioned functional groups [146].

Noticeable differences were also observed in the case of the peak at 933 cm^−1^ attributed to the C-O stretching vibration of the alcohol group, which disappeared in the case of the Zn-treated biomass and shifted to 964 cm^−1^ in the case of the Pb-treated biomass. Also, a new peak was observed at 777 cm^−1^ which is ascribed to the C-H bending vibration, indicating interactions with the carboxyl group. The decrease in the intensity of the peak at 529 cm^−1^ to 520 cm^−1^ and 516 cm^−1^ in the presence of Zn^2+^ and Pb^2+^, respectively, as well as the appearance of a new peak at 467 cm^−1^ associated with the S-S stretching vibration, indicate interactions with the disulfide group [147].

FTIR analysis confirmed the involvement of carboxyl, hydroxyl, carbonyl, and amide I and II bands in the metal uptake by the surface of the bacterial cell, with interactions between Pb^2+^ and Zn^2+^ being observed by the appearance of specific bands in the spectral regions of 2850–2875 cm^−1^, 1150 cm^−1^, 770 cm^−1^, and 460 cm^−1^, as well as the displacement of the peak at 933 cm^−1^ and the decrease in intensity of the vibration of several peaks attributed to the aforementioned functional groups. Noticeable modifications were observed for both the Zn-treated biomass as well as the Pb-treated biomass, suggesting the effect exerted by the contact with the metal ions onto the surface of the cell wall, which is similar to the observations reported by Liaqat et al. [148].

Figure S10 displays the FTIR spectra of *Trichoderma longibrachiatum* biomass prior to and following the treatment with Cr, Pb, and Zn. The absorption bands indicated three primary functional groups: amino, carbonyl, and amide. Differences in the intensity and shape of the peaks could be observed between the control and the metal-treated samples. The broad signal at 3300 cm^−1^, corresponding to stretching vibrations of O-H and N-H bonds from the carboxylic group, and the hydroxyl group decreased to 3277 cm^−1^ in the presence of Cr and Zn, respectively, and to 3280 cm^−1^ in the presence of Pb. The bands at 2923 cm^−1^ and 2853 cm^−1^ are ascribed to the C-H symmetric and asymmetric stretching vibrations of the methylene group [149]. A decrease in signal intensity was observed for the Zn and Pb-treated biomass compared to the control sample. Furthermore, changes in shape and position of the peaks were observed in the presence of the metallic ions. The relative intensity of the C=O stretching vibration band at 1744 cm^−1^, attributed to the ester group in lipids, decreased in the samples treated with Cr, Pb, and Zn. The amide I band at 1649 cm^−1^ suffered a decrease in intensity between 1635 cm^−1^ and 1637 cm^−1^ as a result of the contact with metallic ions. On the other hand, no differences in intensity could be observed for the amide II band at 1545 cm^−1^ corresponding to the stretching vibrations of C=O bonds [150]. In the fingerprint region, slight modifications in the intensity of the vibrations could be observed for the Zn-treated biomass, but the more significant differences were noticed for the Cr and Pb-treated biomass. In the case of the former, a new band appeared at 1406 cm^−1^, attributed to the C=O symmetric stretching vibration of the COO-group. In the case of the Pb-treated biomass, the disappearance of the bands at 1315 cm^−1^ and 1236 cm^−1^ was detected, attributed to the α-helices and ß-sheets of the amide III band owing to the combination of CH stretching and NH bending vibrations [151]. A new band was observed between 1073 cm^−1^ and 1076 cm^−1^ for the samples treated with Pb and Zn, which indicates the interaction of the metal ions with the phosphate and sulfoxide groups. Also, in the case of the Zn-treated sample, the disappearance of the band at 888 cm^−1^ ascribed to the aromatic C-H stretching vibration was observedThe shifting of the band from 558 cm^−1^ to 520–528 cm^−1^ for the metal-treated samples may be from the formation of C-S groups [152].

FTIR analysis revealed modifications in vibrational frequencies on the surface of *T. longibrachiatum* biomass following treatment with Cr, Pb, and Zn, proving the biosorption activity of the cell wall. The biosorption mechanisms were mainly based on physicochemical interactions between the metal ions and the functional groups, with the most noticeable differences being observed in the spectral regions of 2930–2850 cm^−1^, 1740 cm^−1^, 1649 cm^−1^, 1450–1200 cm^−1^ and 550 cm^−1^, which are attributed to the functional groups of lipids, proteins, and carbohydrates [153].

## 4. Conclusions

In this study, a number of sixteen metal-tolerant bacteria and fungi were isolated from soil contaminated with Cr, Pb, and Zn, belonging to the phyla *Firmicutes*, *Proteobacteria*, *Ascomycota*, *Zygomycota*, and *Oomycota*. Zn and Cr presented a high bacteriostatic effect on the isolated bacterial strains, whereas Cr exerted strong fungistatic effects on the fungal strains. The microorganisms studied displayed tolerance at various levels in the following order: Pb > Zn > Cr. *Bacillus marisflavi* and *Trichoderma longibrachiatum* exhibited tolerances above the moderate level for all three metals tested. Innovative electrochemical sensors modified with different materials were developed for the detection of differences in the concentrations of heavy metals prior to and following microbial remediation. 

Good accuracy for the determination of heavy metal contents in real samples was achieved with nanomaterial-based electrochemical sensors. Simple and fast determination of chromium, lead, and zinc was performed by cyclic voltammetry and amperometry using screen-printed carbon or gold electrodes modified with redox mediators, Prussian Blue, or nanocomposites materials. High sensitivities and good reproducibility were achieved in the detection of targeted heavy metals, allowing a sensitive and selective determination of their content in the real samples. 

The bioremediation assay coupled with electrochemical detection revealed the higher removal efficiency of *T. longibrachiatum* compared to *B. marisflavi* for Cr and Zn. The concentration of Cr was reduced by more than 85% in solution, followed by Zn with a removal of 67%. On the other hand, *B. marisflavi* reduced 87% of the concentration of Pb, exhibiting higher efficiency compared to *T. longibrachiatum*, which only reduced 48% of the concentration of Pb in solution. 

Further analyses indicated the mechanisms of action of both strains in contact with metal salts, individually. SEM micrographs indicated morphological alterations as a result of heavy metal stress and interactions between the metal ions and the microbial cells. EDX spectra confirmed the accumulation of metals on the surface of cells, or the prevalence of intracellular accumulation. FTIR spectra indicate the involvement of carboxyl, phosphate, carbonyl, and disulfide functional groups in the biosorption process. 

The main limitation of bioremediation studies Is the difference between laboratory results and in-field observable effects due to the influence of biotic and abiotic factors on microbial metabolism and the efficiency of xenobiotic removal. In order to improve the remediation process and support ecosystem services for the long term, ecological restoration combined with biological methods can be used. Both *Bacillus* sp. and *Trichoderma* sp. can assist in phytoremediation by reducing the effects of stress induced upon plants as well as enhancing the removal of heavy metals from the environment. Environmental biotechnology is a continuously evolving area of research; therefore, it is necessary to gain a better understanding of the most promising microbial strains and develop methods to improve, control, and monitor the remediation potential of indigenous strains for in situ applications.

## Figures and Tables

**Table 1:** Concentration of Cr, Pb, and Zn (mg/kg dw) in the soil samples collected from the contaminated area.

Metals	542 F—D1	543 F—D2	544 F—D3	545 F—D4
Cr (mg/kg dw)	170 ± 0.01	81.1 ± 0.02	80.7 ± 0.02	75.5 ± 0.01
Pb (mg/kg dw)	<4.0 * ± 0.01	41.7 ± 0.09	211 ± 0.05	<4.0 * ± 0.01
Zn (mg/kg dw)	129 ± 0.05	403 ± 0.07	235 ± 0.05	108 ± 0.03

* Quantification limit of the detection method.

**Table 2:** Minimum inhibitory concentration of the bacterial and fungal strains to Cr, Pb, and Zn in the form of K_2_Cr_2_O_7_, Pb (NO_3_)_2_, and ZnSO_4_.

Microbial Strain	Cr (mg/L)	Pb (mg/L)	Zn (mg/L)
*Solibacillus silvestris*	600	800	200
*Bacillus megaterium*	200	200	200
*Bacillus marisflavi*	800	200	200
*Bacillus cereus*	200	200	200
*Paenibacillus pabuli*	200	200	400
*Achromobacter* sp.	600	200	600
*Bacillus subtilis*	200	200	800
*Phoma glomerata*	200	400	600
*Trichoderma citrinoviride*	600	400	1000
*Fusarium fujikuroi*	400	600	600
*Geotrichum candidum*	400	600	800
*Phytophthora* sp.	600	400	1000
*Hypocrea jecorina*	800	1000	1000
*Trichoderma longibrachiatum*	800	800	800
*Mucor* sp.	800	600	1000
*Aspergillus niger*	600	600	1000

**Table 3:** The determination of Cr^6+^, Pb^2+^, and Zn^2+^ concentrations in soil samples using electrochemical sensors and the bioremediation capacity (%) of the bacteria (*B. marisflavi*) and fungal species (*T. longibrachiatum*).

	*B. marisflavi*			*T. longibrachiatum*		
	Metal Concentration (mM)					
	Chromium	Lead	Zinc	Chromium	Lead	Zinc
Before bioremediation	42.2	1.716	4.308	37.5	2.544	9.762
After bioremediation	39.31	0.228	3.584	4.95	1.320	3.224
Decrease in metal concentration (%)	6.85	86.71	16.8	87.5	48.11	66.98

## Data Availability

The data presented in this study are available in this article and Supplementary Materials.

## Supplementary Materials

The following supporting information can be downloaded at: https://www.mdpi.com/article/10.3390/jox14010004/s1, Figure S1: Cyclic voltammograms recorded on the surface of the PB/SPE sensor: in the absence and in the presence of Cr^6+^ at different concentrations; Figure S2: Cyclic voltammograms recorded at the surface of the AuNPs/CS/SPEs sensor in the absence and in the presence of Pb^2+^ at different concentrations; Figure S3: Detection of Zn^2+^ using the MWCNTs-CS/PB/AuSPE sensor in cyclic voltammetry: (A) Voltammograms obtained in the electrolyte solution in the absence and in the presence of different concentrations of Zn^2+^; (B) Calibration curve for the variation of the reduction peak current in cyclic voltammetry vs. concentration of Zn^2+^; Figure S4: Calibration curve obtained for Cr^6+^ ion detection using the PB/SPE sensor (applied potential 0.3 V vs. Ag/AgCl, 0.1 M KCl + 0.1 M HCl). Inset: representation of current intensity dependence on Cr^6+^ concentration; Figure S5: Stability of sensors modified with Prussian Blue for the determination of 1 mM Cr^6+^; Figure S6: Amperometric detection of Pb^2+^ ions using the AuNPs-CS/SPE sensor (E = −0.45 V vs. Ag/AgCl, 0.1 M Tris-HCl, pH 5). Inset: Representation of current intensity dependence on Pb^2+^ concentration; Figure S7: Scanning electron micrographs (SEM) and EDX spectra of *Bacillus marisflavi* biomass (A) control, (B) Pb treated, (C) Zn treated; Figure S8: Scanning electron micrographs (SEM) and EDX spectra of *Trichoderma longibrachiatum* biomass (A) control, (B) Cr treated, (C) Pb treated, (D) Zn treated; Figure S9: Fourier transform infrared spectroscopy spectra of *Bacillus marisflavi* biomass before and after treatment with Pb (NO_3_)_2_ and ZnSO_4_; Figure S10: Fourier transform infrared spectroscopy spectra of *Trichoderma longibrachiatum* biomass before and after treatment with K_2_Cr_2_O_7_, Pb (NO_3_)_2_ and ZnSO_4_; Table S1: Optimization of the working potential and the performance parameters for the amperometric detection of Cr^6+^ using Prussian Blue modified SPEs.
