# Supplementary material for: Microbial Removal of Heavy Metals from Contaminated Environments Using Metal-Resistant Indigenous Strains
Source: J Xenobiot. 2023 Dec 29;14(1):51–78. doi: 10.3390/jox14010004 (PMC10801475; doi:10.3390/jox14010004)
Supplement: Supplementary file 1 [file jox-14-00004-s001.zip › jox-2747579-supplementary.pdf]

Microbial Removal of Heavy Metals from Contaminated Environments Using Metal-Resistant Indigenous Strains

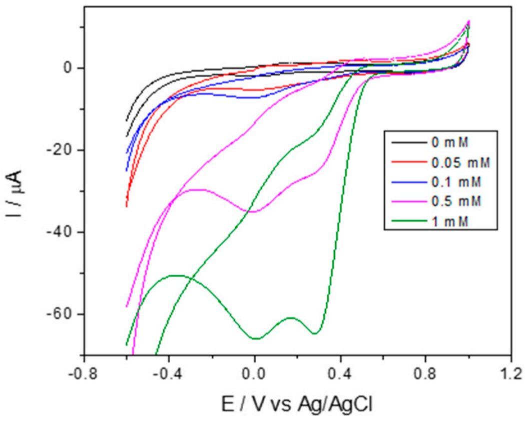

Figure S1. Cyclic voltammograms recorded on the surface of the PB/SPE sensor: in the absence and in the presence of Cr^{6+} at different concentrations (0.1 M KCl + 0.1 M HCl, v = 0.1 V/s).

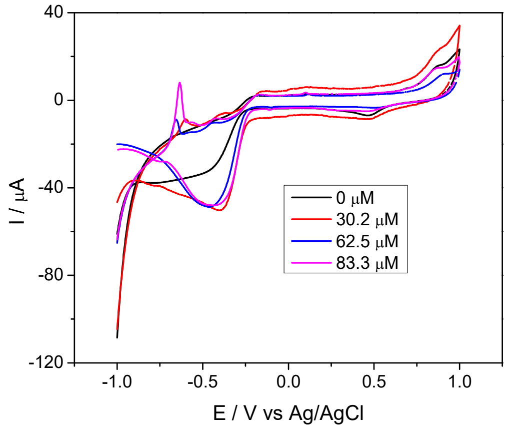

Figure S2. Cyclic voltammograms recorded at the surface of the AuNPs/CS/SPEs sensor in the absence and in the presence of Pb^{2+} at different concentrations (Tris-HCl buffer 0.1 M, pH 5, v = 0.1 V/s).

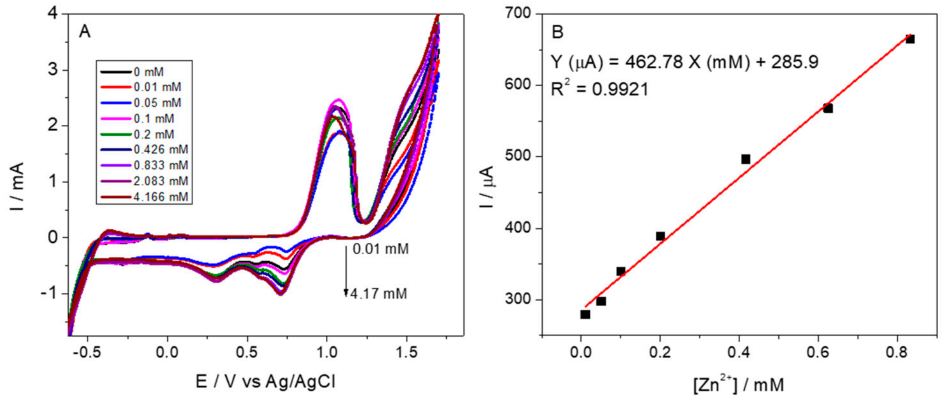

Figure S3. Detection of Zn^{2+} using the MWCNTs-CS/PB/AuSPE sensor in cyclic voltammetry: (A) Voltammograms obtained in the electrolyte solution in the absence and in the presence of different concentrations of Zn^{2+} (v = 0.1 V/s); (B) Calibration curve for the variation of the reduction peak current in cyclic voltammetry vs. concentration of Zn^{2+}.

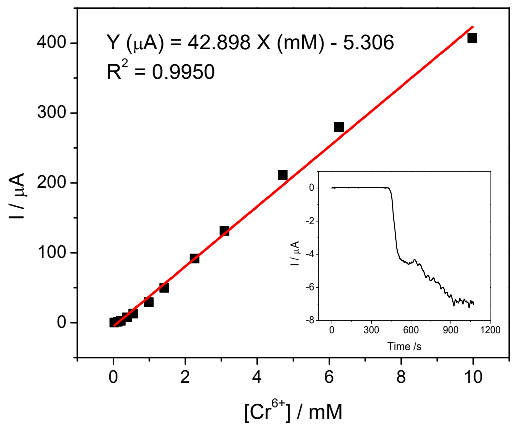

Figure S4. Calibration curve obtained for Cr^{6+} ion detection using the PB/SPE sensor (applied potential 0.3 V vs. Ag/AgCl, 0.1 M KCl + 0.1 M HCl). Inset: Representation of current intensity dependence on Cr^{6+} concentration.

Table S1. Optimization of the working potential and the performance parameters for the amperometric detection of Cr^{6+} using Prussian Blue modified SPEs (0.1 M KCl + 0.1 M HCl).

E (V)	Linear Range (mM)	R^2	Sensitivity (μA/mM)	LOD (μM)	Specific sensitivity ($mA \cdot M^{-1} \cdot cm^{-2}$)
0.28		0.998	39.10	0.61	311.32
0.3	0.035-13.413	0.995	42.90	0.56	341.54
0.33		0.999	36.66	0.82	291.89

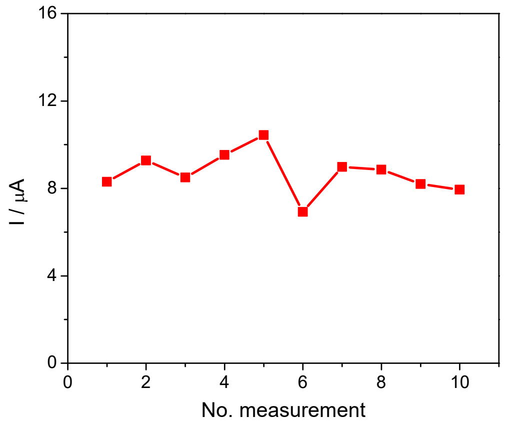

Figure S5. Stability of sensors modified with Prussian Blue for the determination of 1 mM Cr^{6+}.

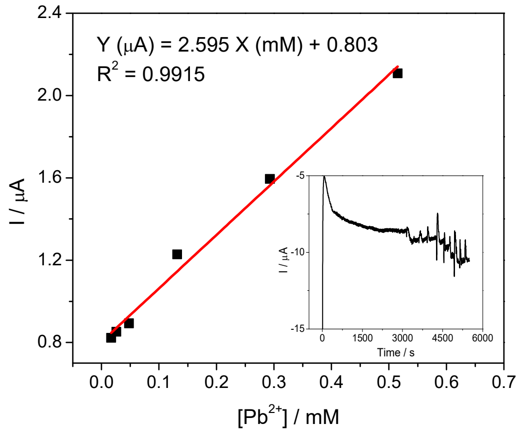

Figure S6. Amperometric detection of Pb^{2+} ion using AuNPs-CS/SPE sensor (E= -0.45 V vs. Ag/AgCl, 0.1 M Tris-HCl, pH 5). Inset: Representation of current intensity dependence on Pb^{2+} concentration.

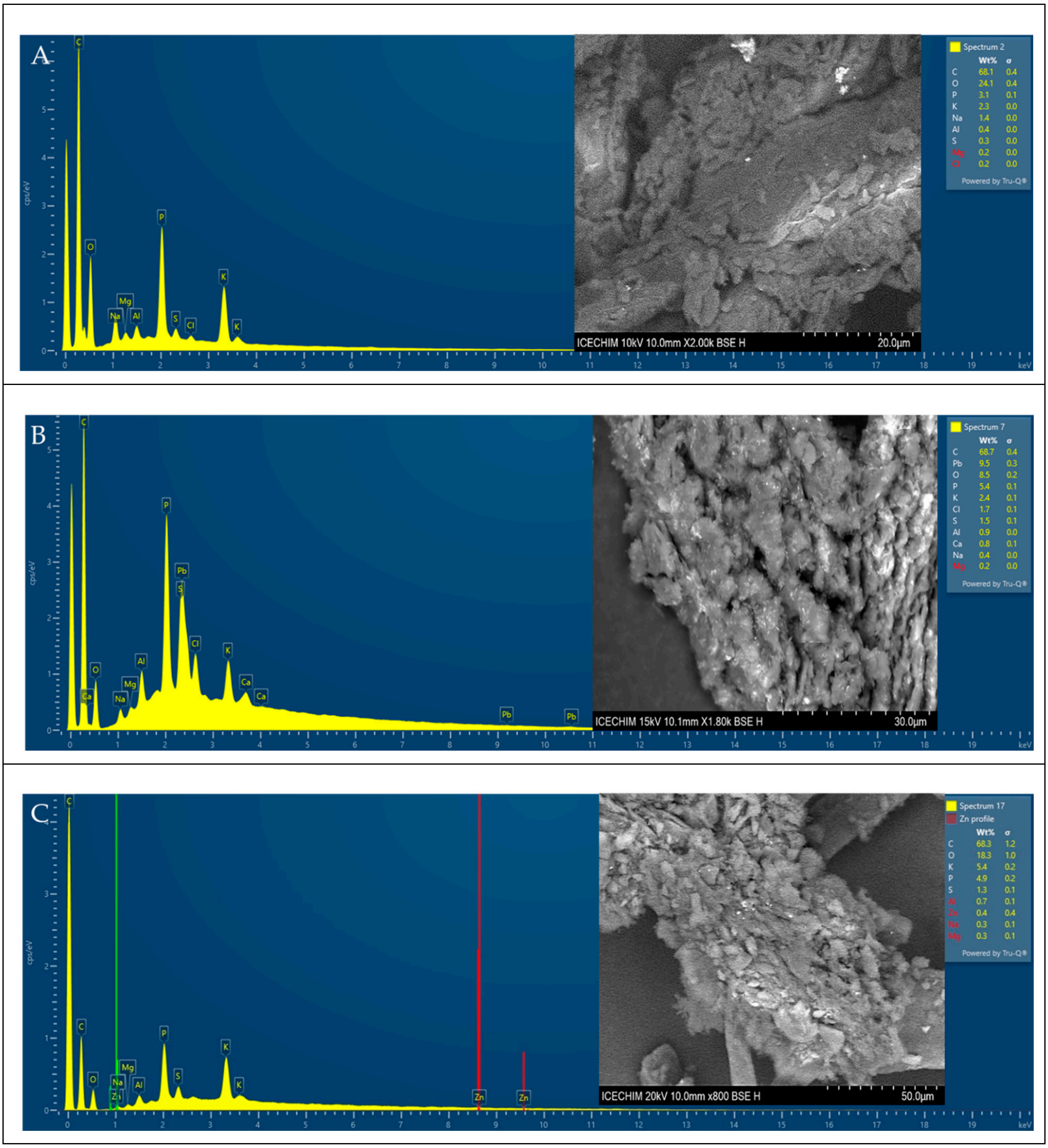

Figure S7. Scanning electron micrographs (SEM) and EDX spectra of *Bacillus marisflavi* biomass A) control, B) Pb treated, C) Zn treated.

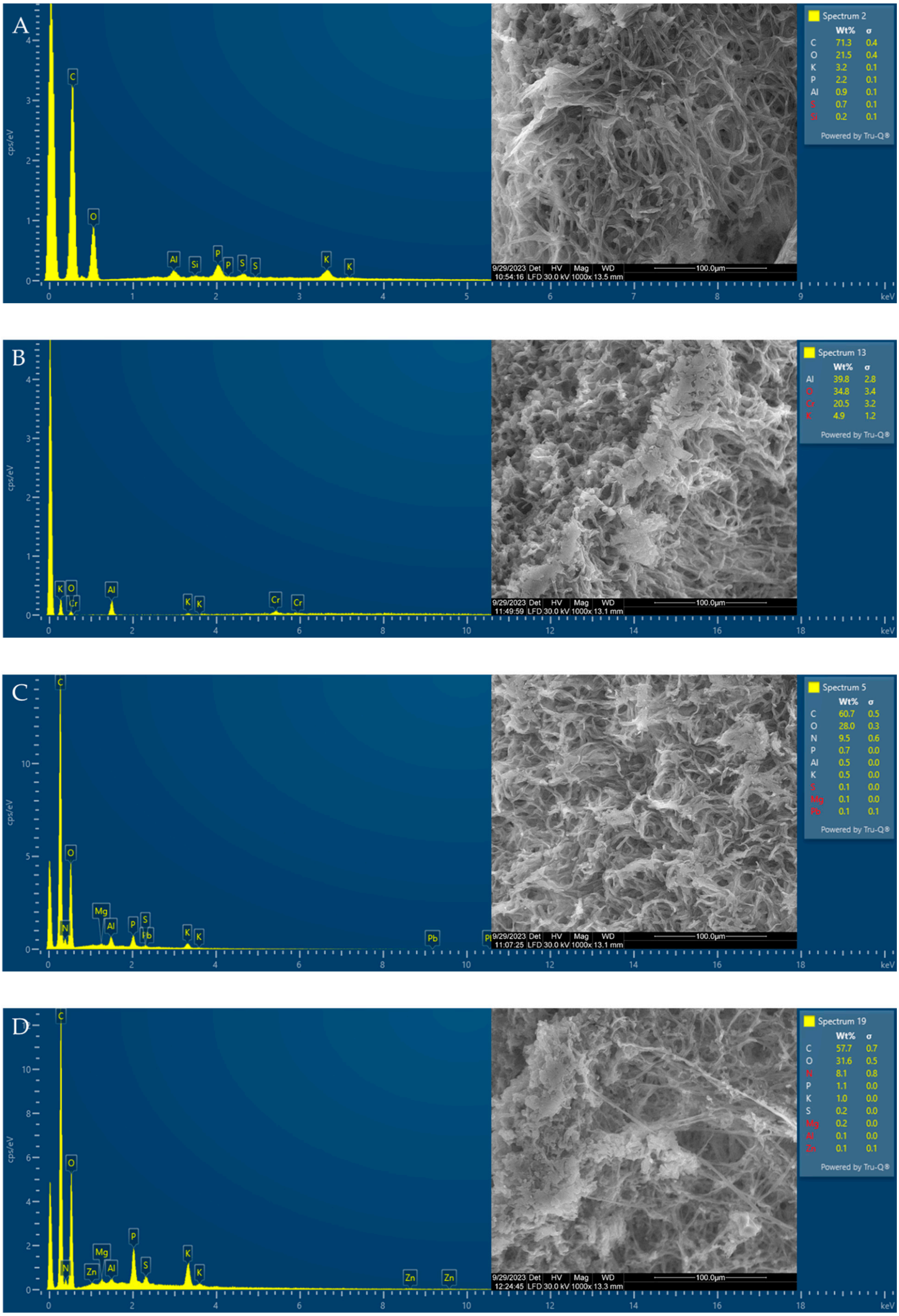

A
cps/eV
keV
Spectrum 2
Wt% σ
C 71.3 0.4
O 21.5 0.4
K 3.2 0.1
P 2.2 0.1
Al 0.9 0.1
S 0.7 0.1
Si 0.2 0.1
Powered by Tru-Q®
9/29/2023 Det HV Mag WD
10:54:16 LFD 30.0 kV 1000x 13.5 mm
100.0μm
B
Spectrum 13
Wt% σ
Al 39.8 2.8
O 34.8 3.4
Cr 20.5 3.2
K 4.9 1.2
Powered by Tru-Q®
9/29/2023 Det HV Mag WD
11:49:59 LFD 30.0 kV 1000x 13.1 mm
100.0μm
C
Spectrum 5
Wt% σ
C 60.7 0.5
O 28.0 0.3
N 9.5 0.6
P 0.7 0.0
Al 0.5 0.0
K 0.5 0.0
S 0.1 0.0
Mg 0.1 0.0
Pb 0.1 0.1
Powered by Tru-Q®
9/29/2023 Det HV Mag WD
11:07:25 LFD 30.0 kV 1000x 13.1 mm
100.0μm
D
Spectrum 19
Wt% σ
C 57.7 0.7
O 31.6 0.5
N 8.1 0.8
P 1.1 0.0
K 1.0 0.0
S 0.2 0.0
Mg 0.2 0.0
Al 0.1 0.0
Zn 0.1 0.1
Powered by Tru-Q®
9/29/2023 Det HV Mag WD
12:24:45 LFD 30.0 kV 1000x 13.3 mm
100.0μm

Figure S8. Scanning electron micrographs (SEM) and EDX spectra of *Trichoderma longibrachiatum* biomass A) control, B) Cr treated, C) Pb treated, D) Zn treated.

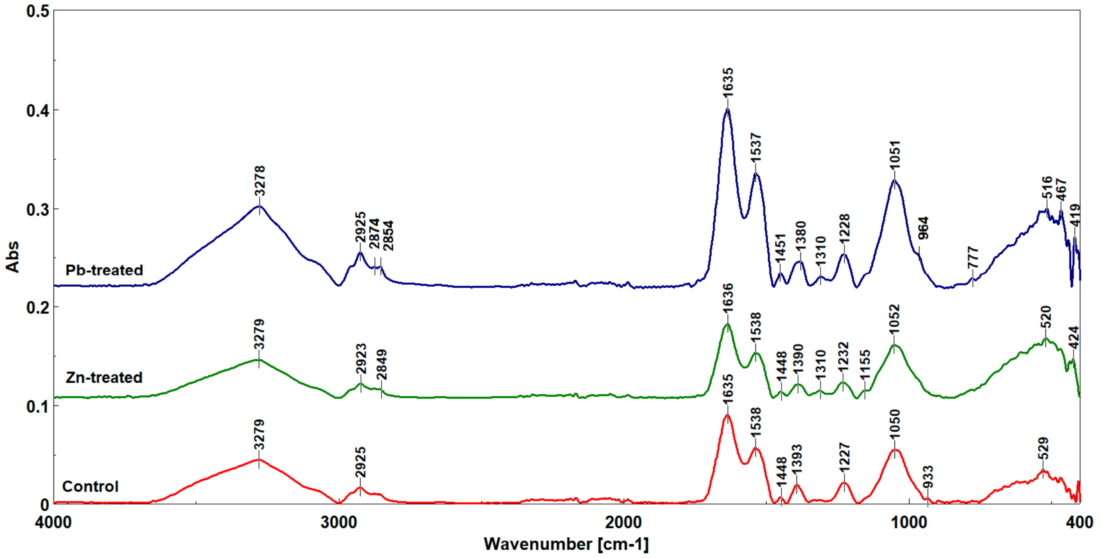

Figure S9. Fourier transform infrared spectroscopy spectra of *Bacillus marisflavi* biomass before and after treatment with Pb $(NO_3)_2$ and $ZnSO_4$.

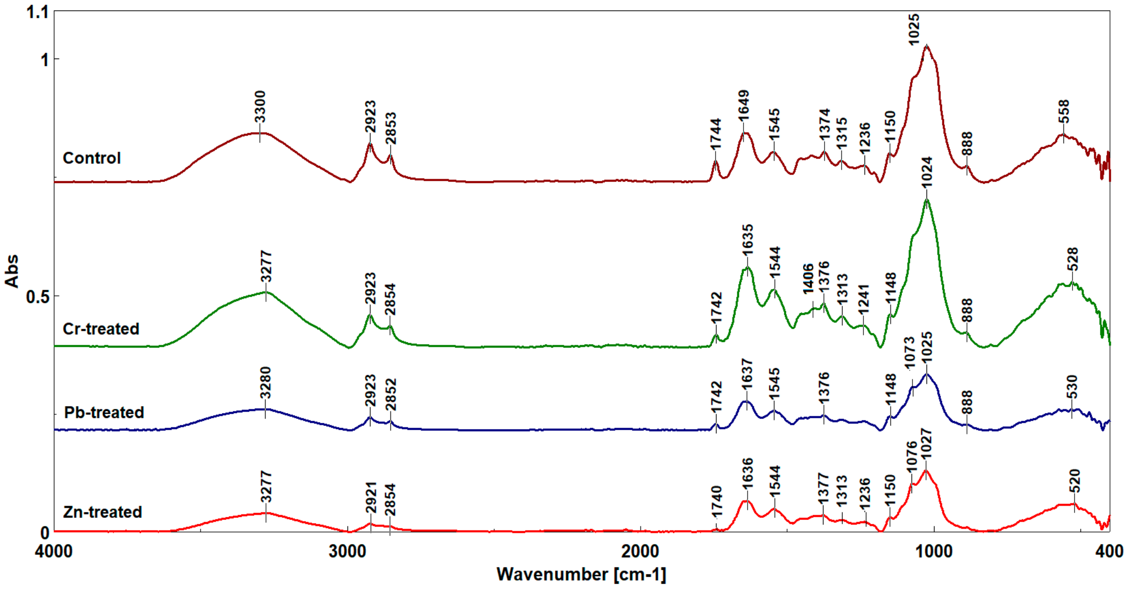

Figure S10. Fourier transform infrared spectroscopy spectra of *Trichoderma longibrachiatum* biomass before and after treatment with $K_2Cr_2O_7$, Pb $(NO_3)_2$ and $ZnSO_4$.
